# Safety of two-year caloric restriction in non-obese healthy individuals

**DOI:** 10.18632/oncotarget.8093

**Published:** 2016-03-15

**Authors:** Sergei V. Romashkan, Sai Krupa Das, Dennis T. Villareal, Eric Ravussin, Leanne M. Redman, James Rochon, Manjushri Bhapkar, William E. Kraus, for the CALERIE Study Group

**Affiliations:** ^1^ National Institute on Aging, Bethesda, MD, USA; ^2^ Jean Mayer US Department of Agriculture Human Nutrition Research Center on Aging at Tufts University, Boston, MA, USA; ^3^ Washington University School of Medicine, St Louis, MO, USA; ^4^ Baylor College of Medicine and Michael E DeBakey VA Medical Center, Houston, TX, USA; ^5^ Pennington Biomedical Research Center, Baton Rouge, LA, USA; ^6^ Rho Federal Systems, Chapel Hill, NC, USA; ^7^ Duke Clinical Research Institute and Duke University School of Medicine, Durham, NC, USA

**Keywords:** calorie restriction, safety, humans, dietary energy restriction, Gerotarget

## Abstract

**Background:**

The extent to which sustained caloric restriction (CR) in healthy non-obese adults is safe has not been previously investigated.

**Objective:**

Assess the safety and tolerability of sustained two-year CR intervention in healthy, non-obese adults.

**Design:**

A multi-center, randomized controlled trial. Participants were randomized using a 2:1 allocation in favor of 25% CR vs. Ad-Libitum intake (AL). Adverse and serious adverse events (AE, SAE), safety laboratory tests, and other safety parameters were closely monitored.

**Results:**

Three participants were withdrawn from the CR intervention because of the safety concerns. No deaths and one SAE was reported by participants in the CR group. Although the difference in AE between AL and CR groups was not significant, within the CR group, the incidence of nervous system (*p* = 0.02), musculoskeletal (*p* = 0.02) and reproductive system (*p* = 0.002) disorders was significantly higher in the normal-weight than in the overweight participants. At months 12 and 24, bone mineral densities at the lumbar spine, total hip, and femoral neck of participants in the CR group were significantly lower than in those in the AL group.

**Conclusions:**

Two-years of CR at levels achieved in CALERIE was safe and well tolerated. Close monitoring for excessive bone loss and anemia is important.

## INTRODUCTION

Numerous studies have shown that caloric restriction (CR) increases mean and maximum lifespan in many, but not all models [1-3]. In many rodent strains, non-human primates, and humans, CR decreases the risk for and delays the onset of diseases and conditions associated with aging, including diabetes, cardiovascular disease and cancer [3-5]. The overall aim of the recently completed Comprehensive Assessment of Long-Term Effects of Reducing Intake of Energy (CALERIE) study was to test the hypothesis that sustained for two years caloric restriction in healthy men aged 21 to 50 and healthy women aged 21 to 47 will result in adaptive changes analogous to those that occur in rodents subjected to CR. The adaptive responses thought to be involved in slowing the aging process and protecting against age-related diseases were of particular interest. Effects of two years of sustained CR on primary, secondary and a number of related outcomes are reported separately [6]. As the extent to which sustained CR in healthy non-obese adults might be successful but accompanied by unacceptable adverse effects has not been previously investigated. We here report on one of the secondary objectives of CALERIE, which was to systematically examine the safety of CR intervention in non-obese healthy adults.

## RESULTS

### Achieved levels of caloric restriction

As described [6], on average, in an intent-to-treat analysis, daily energy intake declined from baseline in CR group by 480 kcal/d during the first six months of intervention, then stabilized at approximately 234 kcal/d below baseline for the remainder of the trial, resulting in CR averaging 11.9% over two years (19.5±0.8% during the first six months and 9.1±0.7% on average for the remainder of the study, p<.0001 vs. AL).

### Study and intervention discontinuations

The baseline characteristics of 218 participants included in the safety analyses are summarized in Table 1 and details on screening, recruitment and retention are published elsewhere [6, 7]. A total of 30 participants (26 in the CR and 4 in the AL group) discontinued from their respective interventions prior to completing the study. The 26 CR participants included three women who became pregnant during the study, three withdrawn for safety reasons (see below), 14 who withdrew consent or discontinued participation for other reasons, and six participants relocated prior to study completion. Three participants in the CR group were permanently discontinued from the intervention but completed all follow-up evaluations and were included in the safety analysis. In the control group, three women became pregnant, one participant withdrew consent, and all four were permanently discontinued. Those who failed to provide complete information tended to be younger and better educated (*p* = 0.01 for both); otherwise, there were no significant differences with respect to demographic and other baseline characteristics [6].

**Table 1:** Demographic, anthropometric and clinical characteristics at baseline for the 218 participants who are included in safety analysis

	Males (*n*= 66)		Females (*n*= 152)		Overall (*n* = 218)	
Characteristic	AL (*n* = 22)	CR (*n* = 44)	AL (*n* = 53)	CR (*n* = 99)	AL (*n* = 75)	CR (*n* = 143)
**Age, y**	37.8 (7.1)^1^	40.5 (7.2)	37.9 (6.9)	36.8 (7.2)	37.9 (7.0)	38.0 (7.3)
**Race:**						
**White**	18 (81.8%)^2^	37 (84.1%)	39 (73.6%)	74 (74.7%)	57 (76.0%)	111 (77.6%)
**African-American**	1 (4.6%)	2 (4.6%)	10 (18.9%)	13 (13.1%)	11 (14.7%)	15 (10.5%)
**Other**	3 (13.6%)	5 (11.4%)	4 (7.6%)	12 (12.1%)	7 (9.3%)	17 (11.9%)
**Height, m**	176.7 (5.3)	177.1 (7.2)	165.0 (6.8)	165.2 (6.4)	168.4 (8.3)	168.9 (8.6)
**Baseline Weight, kg**	79.8 (6.6)	81.6 (8.3)	68.0 (6.9)	67.7 (6.3)	71.5 (8.7)	72.0 (9.5)
**Baseline BMI, kg/m^2^**	25.6 (1.7)	26.0 (1.6)	24.9 (1.6)	24.8 (1.7)	25.1 (1.6)	25.2 (1.8)
**Body Fat, %**	25.7 (4.0)	26.1 (3.1)	36.8 (4.2)	36.0 (4.3)	33.6 (6.6)	32.9 (6.1)
**FFM, kg**	59.3 (5.2)	60.3 (6.0)	42.8 (3.6)	43.2 (4.1)	47.6 (8.6)	48.5 (9.2)
**FM, kg**	20.5 (3.9)	21.3 (3.7)	25.2 (4.8)	24.4 (4.3)	23.8 (5.0)	23.5 (4.3)
**Waist Circumference, cm**	88.5 (5.5)	89.0 (5.5)	78.3 (5.5)	77.0 (5.5)	81.3 (7.2)	80.7 (7.8)
**Systolic BP, mmHg**	117.9 (7.6)	116.2 (8.2)	108.4 (9.4)	110.3 (10.1)	111.2 (9.9)	112.1 (9.9)
**Diastolic BP, mmHg**	73.2 (7.6)	73.6 (7.5)	70.4 (6.8)	71.4 (7.5)	71.2 (7.1)	72.1 (7.5)
**Contraception:**						
**Tubal ligation**	-	-	5 (9.4%)^2^	8 (8.1%)	5 (9.4%)	8 (8.1%)
**Hysterectomy**	-	-	2 (3.8%)	5 (5.1%)	2 (3.8%)	5 (5.1%)
**Oral**	-	-	10 (18.9%)	23 (23.2%)	10 (18.9%)	23 (23.2%)
**Barrier/Condom**	-	-	11 (20.8%)	16 (16.2%)	11 (20.8%)	16 (16.2%)
**Other**^3^	-	-	17 (32%)	30 (30.3%)	17 (32%)	30 (30.3%)
**None/Unknown**	-	-	8 (15%)	17 (17.2%)	8 (15%)	17 (17.2%)
**Bone mineral density, z-score**						
Lumbar spine	0.1 ± 1.0	−0.0±1.0	−0.7±1.7	−0.3±1.0	−0.5 ± 1.5	−0.2 ± 1.0
Total hip	0.2 ±0.8	−0.1±0.8	−0.1±0.8	−0.0±0.9	−0.0 ± 0.8	−0.0 ± 0.8
Femoral neck	0.1± 0.9	−0.1±0.9	−0.3±0.9	−0.2±0.9	−0.2 ± 0.9	−0.2 ± 0.9

1 Mean (s.d.)

2 *n* (%)

AL = *Ad libitum* treatment group; CR = Caloric Restricted treatment group; s.d. = standard deviation; FFM = fat free mass; FM = fat mass, BP = blood pressure

3 Includes contraceptive vaginal ring “NuvaRing™,” barrier method plus spermicide, intrauterine device, spousal vasectomy, abstinence, and natural family planning

### Adverse events and serious adverse events

Overall, a total of 3,332 adverse events were reported in the study. In the CR group, 95.1% of participants reported a total of 1,995 events and a total of 1,337 events were reported by 96.0% of participants enrolled in the AL group. Seven participants reported a total of eight serious adverse events during the study. Of these, one event (spontaneous miscarriage) was reported by a participant in the CR group and seven (pneumothorax, uterine leiomyoma, scoliosis, spinal osteoarthritis, intestinal abscess, small intestinal obstruction, and atrial septal defect) were reported by participants in the AL group. All AL participants who experienced the SAEs completed the study.

In 11 MedDRA System Organ Classes (SOCs), 10% or more of participants in either group experienced at least one non-serious adverse event. These SOCs included infections and infestations; nervous system disorders; psychiatric disorders; musculoskeletal and connective tissue disorders; gastrointestinal disorders; general disorders; respiratory, thoracic and mediastinal disorders; reproductive disorders; injury, poisoning and procedural complications; immune system disorders; and skin and subcutaneous tissue disorders. Only reproductive system disorders (24.5% CR vs. 14.7% AL) and skin disorders (15.4% CR vs. 10.7% AL) were reported more frequently by CR participants. However, differences between the treatment groups were not statistically significant (*p*=0.12 and *p*=0.41, respectively). A total of 28 specific AEs in 10 MedDRA organ classes were reported by 10% or more of participants in either treatment group. Among AE's that were reported in 5% or more of participants in either treatment group, the incidence of 13 individual AEs in 8 MedDRA organ classes was higher in the CR than in the AL group, but none of the differences between the treatment arms reached statistical significance (Table 2).

**Table 2:** Incidence and number of non-serious adverse events reported more frequently by participants in the CR group, and in at least 5% of CR participants

Preferred Term	Ad Libitum (*n* = 75)		Caloric restriction (*n* = 143)		*p*-value^3^
	% (pts)^1^	No. events^2^	% (pts)^1^	No. events^2^	
**Sinus congestion**	10.7	17	16.1	43	0.31
**Dysmenorrhea**	15.1	31	21.2	78	0.40
**Dizziness**	12.0	14	14.7	48	0.68
**Diarrhea**	13.3	16	14.0	27	1.00
**Nasal congestion**	6.7	14	13.3	28	0.17
**Constipation**	6.7	11	12.6	44	0.25
**Toothache**	8.0	12	11.9	21	0.49
**Muscle strain**	4.0	4	10.5	16	0.12
**Gastroenteritis viral**	6.7	5	9.1	16	0.61
**Pain**	6.7	10	8.4	20	0.79
**Rash**	1.3	1	6.3	9	0.17
**Urinary tract infection**	5.3	6	5.6	11	1.00

1 Percent of participants who experienced at least one event

2 Total number of AEs (includes multiple events from the same participant)

3 Fisher's exact test comparing the incidence (proportion of patients) reporting at least one adverse events between the two treatment groups at specific preferred term level only

**Figure 1:**
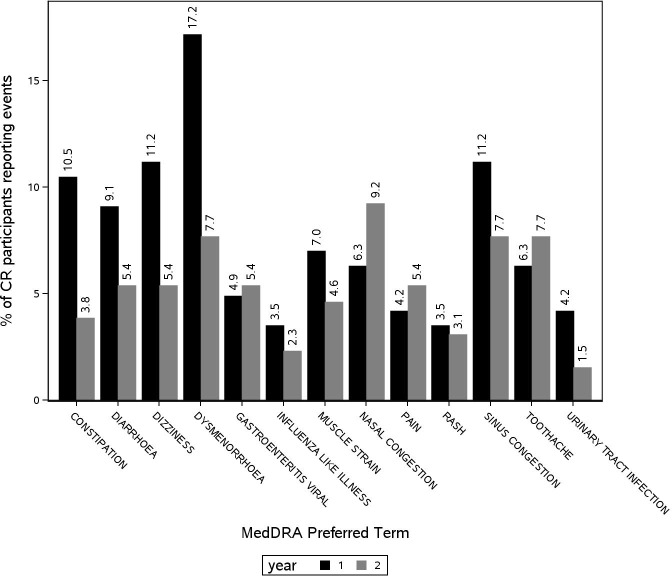
Individual adverse events in CR participants in years 1 and 2 of CALERIE Includes only AEs that occurred more often in the CR arm and were reported by at least 5% of CR participants.

Of interest is the incidence of AEs in the CR group by BMI and duration of the intervention. In the CR group, in all 11 MedDRA SOCs which occurred in 10% or more of participants in either group, incidence of adverse events was higher in normal-weight than in overweight participants (Table 3). For nervous, musculoskeletal and reproductive system disorders, the incidence was 20 percentage points higher in normal-weight participants. These differences were statistically significant (p=0.016, p=0.014 and p=0.001, respectively). In the control group, only incidence of immune system disorders was 20 percentage points higher in overweight than in normal-weight participants (31.6% and 10.8%, respectively, p=.047). In both CR and control groups, a higher proportion of normal weight than overweight women reported dysmenorrhea (25.9% vs 14.6% and 18.5% vs 9.5%m respectively). Among the individual events which occurred more often in the CR group than the AL group, and in at least 5% of the CR subjects, the incidence of eight out of 13 individual events was noticeably higher in the first year of the study with about twice as many participants in the first year reporting dysmenorrhea, dizziness, diarrhea, and constipation (Figure 1). In the control group, reporting pattern was the same with about twice as many participants in the first year reporting dysmenorrhea, dizziness, diarrhea, and constipation (not shown).

**Table 3:** Incidence and number of non-serious adverse events reported by participants in the CR group by system organ class and BMI

	Overall (*n* = 143)		Normal weight (*n* = 68)		Overweight (*n* = 75)		*p*-value^3^
System Organ Class	% (pts)^1^	No. events^2^	% (pts)^1^	No. events^2^	% (pts)^1^	No. events^2^	
**Overall**	95.1	1995	97.1	1164	93.3	831	0.45
**Infections & infestations**	62.2	239	67.6	133	57.3	106	0.23
**Nervous system disorders**	58.7	508	69.1	347	49.3	161	0.02
**Musculoskeletal & connective tissue disorders**	52.4	224	63.2	137	42.7	87	0.02
**Gastrointestinal disorders**	51.7	289	60.3	137	44.0	152	0.07
**General disorders**	48.3	188	52.9	109	44.0	79	0.32
**Respiratory, thoracic & mediastinal disorders**	41.3	178	44.1	97	38.7	81	0.61
**Injury, poisoning & procedural complications**	28.7	67	30.9	33	26.7	34	0.59
**Reproductive disorders**	24.5	133	36.8	77	13.3	56	0.002
**Reproductive disorders (women only)**^4^	34.3	132	41.4	76	24.4	56	0.09
**Immune system disorders**	16.1	47	17.6	26	14.7	21	0.66
**Skin & subcutaneous tissue disorders**	15.4	30	19.1	18	12.0	12	0.26
**Psychiatric disorders**	16.8	49	17.6	25	16.0	24	0.83

1 Percent of participants who experienced at least one event

2 Total number of AEs (includes multiple events from the same participant)

3 Fisher's exact test comparing the incidence (proportion of patients) reporting at least one adverse events between the two BMI strata at system organ class level only

4 *n* = 99 for all, *n* = 58 for normal weight and *n* = 41 for overweight

### Safety laboratory test results

A higher proportion of participants in the CR than in the control group had higher than normal serum creatine kinase levels and mean cell volume during the study. In several participants on several occasions creatine kinase levels increased up to 10 times above the upper limit of normal. Lower than normal levels of the eGFR, total hemoglobin, hematocrit, mean corpuscular hemoglobin, platelet and white blood cell count as well as serum iron and sodium were observed more often in the CR group. However, most of the abnormalities were small, with levels slightly below the lower limit of normal and none of the differences between the treatment arms reached statistical significance (Table 4). Unlike incidence of the AEs which was higher in the normal weight CR participants, incidence of select abnormal laboratory tests was higher in the overweight than in the normal-weight CR participants (not shown). Approximately 45% of the overweight participants experienced increased levels of serum creatine kinase while this abnormality was observed only in 29.4% of the normal-weight participants. Correspondingly, 9.3% and 20.0% of the overweight participants reported decreases in hematocrit and sodium levels vs. 4.4% and 7.4% of the normal-weight participants, respectively. In the control group, a higher proportion of the normal-weight than the overweight participants reported increases in creatine kinase levels (37.8% vs 29%) and decrease in hematocrit levels (11% vs 0%). No difference in levels of sodium was observed between the treatment arms.

**Table 4:** Select laboratory test abnormalities observed more frequently in participants enrolled in the CR group

Laboratory Test	Ad Libitum (*n* = 75)		Caloric restriction (*n* = 143)		*p*-value^3^
	% (pts)^1^	No. occurrences^2^	% (pts)^1^	No. occurrences^2^	
Creatine kinase (high)	33.3	36	37.8	123	0.56
Mean cell volume (high)	14.7	23	18.2	68	0.57
White blood cells (low)	20.0	38	27.3	124	0.25
Sodium (low)	10.7	11	14.0	29	0.53
Iron (low)	5.3	5	9.8	17	0.31
eGFR (low)	4.0	7	6.3	14	0.55
Hemoglobin (low)	4.0	5	7.7	30	0.39
Hematocrit (low)	5.3	6	7.0	23	0.78
Platelet count (low)	2.7	5	5.6	23	0.50
Mean corpuscular hemoglobin	2.7	4	4.9	38	0.72

1 Percent of participants with at least one abnormal laboratory test result above (“high”) the upper limit of normal or below (“low”) the lower limit of normal

2 Total number of occurrences of a specific laboratory test abnormality

3 Fisher's exact test comparing the incidence (proportion of patients) with at least one abnormality between the two treatment groups at specific laboratory test level only

### Electrocardiogram

No clinically significant ECG abnormalities were observed at any time point during the study. The most commonly reported abnormality was bradycardia, which could be considered a favorable effect of caloric restriction due to decreased sympathetic or thyroid tone [8].

### Physical examination and vital signs

There were no clinically significant abnormalities observed during the physical examination nor were there any clinically significant changes in vital signs observed during the study.

### Depression

Five (3.5%) participants in the CR group and 1 participant (1.3%) in the control group had BDI scores suggestive of moderate depression. No BDI scores suggestive of the severe depression were observed. As prescribed in the depression surveillance protocol, all six participants were further evaluated by a licensed professional to confirm or reject the diagnosis. No participants discontinued the CR intervention or the study because of depression.

Eating disorders. There was a screening protocol to exclude any study candidates who displayed signs of eating disorder pathology. During the study, participants were monitored for signs of eating disorders using two questionnaires - Multi-axial Assessment of Eating Disorder Symptoms [9] and Body Acceptability Morph [10]. Ninety-four (65.7%) CR participants who had scores suggestive of eating disorders at some point during the study were administered the Interview for the Diagnosis of Eating Disorders-Fourth Version, but no eating disorders were confirmed and no further actions were taken. Thirty nine (52%) of participants in the control group had scores suggestive of eating disorder at some point during the study.

### Excessive bone loss

At month 12 and 24, the BMD at lumbar spine, total hip and intertrochanter of participants in the CR group was significantly lower than in those in the control group (p<0.001 for the between-group differences at all sites). The BMD at femoral neck and trochanter was also lower in the CR group compared to participants in the control group at month 12 (p=0.0022 and p=0.0023, respectively by site) and month 24 (p=0.034 and p=0.0026, respectively by site) visits. There were no differences in BMD at the whole body and distal radius between the CR group and control group. Because of the excessive bone loss, two CR participants discontinued the intervention temporarily and one other permanently.

### Drop in hematocrit levels

The anemia and hematocrit surveillance protocol was triggered when there was a decrease in hemoglobin, hematocrit, or red blood cells below the lower limit of normal (LLN) or a decrease of 5 percentage points in hematocrit level (even if it stayed within the normal range). There were 99 such episodes among 35 CR participants (24.5%). The hematocrit drop resolved before the end of the study in 18 participants; two participants were withdrawn for treatment-resistant decreases in hematocrit while 7 others withdrew for other reasons; and, in 8 participants, it did not resolve completely but they remained in the study. In the control group, there were 34 episodes among 17 participants (23%) triggering the anemia surveillance protocol.

## DISCUSSION

CALERIE was the first study in healthy non-obese adults between the ages of 21 years and 50 years at screening that developed and implemented a comprehensive surveillance protocol to assess the safety of two years’ sustained caloric restriction. Our results show that sustained caloric restriction at levels achieved in CALERIE was safe and well tolerated. No deaths were observed in the study. One serious adverse event, a miscarriage, was reported in the CR group but its relationship to the intervention was considered doubtful. Only in two MedDRA system organ classes, reproductive system and skin disorders, participants in the CR group reported more non-serious events than participants in the control group, but none of the difference were statistically significant. Correspondingly, none of the differences between the treatment arms for the twelve individual non-serious events that were reported more often by participants in the CR group reached statistical significance. Of interest is the higher incidence of adverse events in normal weight than in overweight participants with differences between the BMI strata reaching statistical significance for the nervous, musculoskeletal and reproductive system disorders. However, because the total number of participants in each BMI strata was small, no definitive inferences about the effects of BMI on the incidence of adverse events could be made and more studies are needed to answer this question. There also were not enough data to conclude that a higher incidence of select adverse events observed in the first year of the study indicated that the potentially negative effects of CR would manifest early after initiation of the intervention. This is confounded, however, with lower adherence to the CR intervention in the second year. Overall, non-serious adverse events reported more often by participants in the CR group appear to be minor and do not pose a safety hazard.

It is well established that weight loss in obese adults is accompanied by loss of BMD: about 10% weight loss results in 1% to 4 % loss of BMD at the hip or lumbar spine [11-14]. CALERIE showed that in younger non-obese adults, weight loss predictably was also accompanied by modest BMD loss at the clinically relevant sites, although the clinical significance of a change in 0.16 T-score units is unclear. Using the World Health Organization's Fracture Risk Assessment tool, we estimated that such degree of BMD loss in a middle-aged non-obese woman would increase her 10-year risk of osteoporotic fracture by about 0.2%. The increase in fracture risk associated with the lowering of BMD in CALERIE appears to be very small, but longer term studies would be needed to fully assess the clinical importance of these findings. Whether this amount of bone loss accompanied by weight loss is physiologically significant is unclear.

Safety laboratory tests showed that CR in healthy non-obese adults at levels achieved in CALERIE increased the risk for significant decreases in hematocrit, which in some participants required iron supplementation or resulted in discontinuation of the CR intervention. Increases in levels of creatine kinase observed in some participants in the CR group could be explained by a significant loss of muscle mass, but data are insufficient to confirm or reject this hypothesis and further studies would be needed.

We also compared the safety laboratory test abnormalities observed in the CALERIE Phase 1 studies with findings from the current CALERIE study. While some of the changes in the safety laboratory tests observed in CALERIE Phase 1 studies were also observed in the current CALERIE study, some were not. At one of the clinical sites participating in CALERIE Phase 1 studies, hyperkalemia was observed in a large proportion of participants, but less than 5% of the CR participants in the current trial developed this abnormality. There were also concerns about CR participants developing hypocalcemia but no cases were observed in the current CALERIE study. In both Phase 1 and Phase 2 (current) CALERIE studies a higher proportion of participants in the CR than in the AL group showed decreases in hemoglobin, hematocrit and serum iron levels.

No eating disorders were diagnosed in the study and no participants discontinued the CR intervention because of depression. A careful screening of the study candidates and exclusion of any who showed the signs of eating disorders or depression could have contributed to this outcome.

Although CALERIE is the largest study to date on the effects of sustained caloric restriction in healthy non-obese adults, the total number of participants remains relatively small, thus the power remains low to make definitive conclusions about the safety of CR in this population can be made. Also, because CALERIE population is highly selective, safety findings from this study cannot be extrapolated to the general population. Another potential limitation is that CALERIE utilized the safety measures that are standard in pharmaceutical trials. It is possible that studies on effects of caloric restriction in humans require monitoring of a different set of safety parameters which are as yet not well-defined. Finally, there data are insufficient to assess the clinical significance of the observed effects of CR on risk factors for diseases associated with aging versus the clinical significance of the observed increases in risk for anemia and excessive bone loss. Based upon available information, we conclude that in future studies of sustained caloric restriction in non-obese healthy individuals close monitoring for excessive BMD loss and anemia is important.

## MATERIALS AND METHODS

### Study design and Intervention

CALERIE was a two year multi-center, parallel-group, randomized controlled trial; the full details have been published [15]. Randomization was stratified by gender and body mass index (BMI) within each clinical center, with BMI dichotomized into normal weight (22.0 to 24.9 kg/m^2^) and overweight (25.0 to 27.9 kg/m^2^). A 2:1 allocation ratio in favor of the CR intervention was applied in order to maximize the number of participants receiving the CR intervention. Given the nature of the study interventions, neither the participants nor the CALERIE intervention staff were blinded to the treatment assignments. However, staff evaluating study endpoints was blinded and communications between the two groups of staff members were minimized. The monitoring of adherence to the intervention was driven by a target trajectory of weight loss reaching 15.5% below baseline after one year of intervention followed by maintenance of this weight over the second year of the study. This trajectory was modeled after CALERIE phase 1 pilot studies data that predicted the expected changes in body weight for participants adherent to a one year 25% CR.

The details of the CR intervention have been reported previously [16] and were chosen to optimize the likelihood that a substantial degree of CR was achieved (estimated to be 25% based upon modeling of Phase I data). A complete daily vitamin and mineral supplement as well as a daily calcium supplement of 1,000 mg were provided to both the intervention and *ad libitum* (AL) control group to ensure that participants met the current recommendations for these nutrients.

### Participants

A total of 238 healthy, non-obese men aged 21 to 50 years and women aged 21 to 47 years of different races provided written informed consent. Of these, 220 participants were randomized and 218 started the interventions. All 218 participants who started the interventions were included in the safety analyses. All study candidates were carefully screened to ensure that they were free of significant medical conditions, eating disorders and depression, did not have a history of alcohol and drug abuse, their prespecified safety laboratory markers were within the normal range, and they were not taking any medications (oral, implantable and vaginal contraceptives were allowed). Study candidates with low bone density (BMD) defined as a BMD of the total hip, femoral neck, or lumbar spine equal to or less than a T-score of −2.3 were excluded from participation. Current smokers and those who quit smoking less than 12 months prior to screening and breast-feeding or pregnant women were excluded. After some initial pregnancies were reported in the trial, inclusion criteria for all women participants required the practice of an effective contraception method. Contraception use was verified at Month 1, 3, 6, 9, 12, 18, and 24 visits. Acceptable forms of contraception included tubal ligation, partial or complete hysterectomy, oral contraceptive pills, implanted progesterone “Implanon™,” contraceptive vaginal ring “NuvaRing™,” barrier method plus spermicide, intrauterine device, spousal vasectomy, abstinence, and natural family planning when other contraceptive methods were prohibited due to religious reasons. Detailed patient characteristics, information on participant recruitment, screening and retention as well as a complete list of eligibility criteria are published elsewhere [6, 7, 15].

### Safety measures

Safety of the participants during CALERIE Phase 2 study was closely monitored by the clinical site physician-investigators, the study Safety Committee, and by the independent Data and Safety Monitoring Board appointed by the National Institute on Aging. A complete medical history was performed during screening and included a review of all major organ systems and all medications taken during a 30-day period prior to screening. A thorough review of reproductive status, contraceptive use and menstrual history for women was performed. An abbreviated medical and medication history was reviewed at baseline. A complete physical examination was performed for each participant at screening, baseline, Months 12, and 24 visits. The vital signs included blood pressure, oral temperature, respiratory rate and pulse rate and were collected at every clinic visit.

CALERIE used the International Conference on Harmonization E2A definitions of adverse event (AE) and serious adverse event (SAE) [17]. Participants were given diaries to record signs, symptoms and adverse events occurring during the study. This included anecdotal reports of medical problems such as constipation, dizziness, fatigue, pain, nausea and infections, and menstrual irregularities for women. In addition to the regularly scheduled clinic visits, participants in both treatment groups were contacted monthly by study staff either by phone or in person to collect the adverse events. Diary-reported AEs were reviewed by the study staff, and newly emerging AEs, as well as the status of any AEs that have not yet resolved, were recorded in the case report forms. During these contacts, the study staff members proactively asked whether any sign, symptom or AEs had occurred since the previous contact. All adverse events were coded using the Medical Dictionary for Regulatory Affairs (MedDRA), version 14.4.

The safety clinical laboratory tests included hematology, clinical chemistry and urinalysis which were performed at a central laboratory at baseline and follow-up visits at months 1, 3, 6, 9, 12, 18 and 24. Hematological tests included white cell count with differential, red cell count (RBC), RBC morphology, hemoglobin, hematocrit, and platelet count. Serum chemistry tests included sodium, potassium, calcium, magnesium, iron, albumin, total protein, total bilirubin, alanine transaminase, aspartate aminotransferase, gamma-glutamyl transpeptidase, alkaline phosphatase, creatine phosphokinase, creatinine, blood urea nitrogen, estimated glomerular filtration rate (eGFR), glucose and uric acid. Total, high density lipoprotein - and calculated low density lipoprotein cholesterol, triglycerides, C-reactive protein, and blood glucose and insulin values were also performed. Urinalysis included pH, protein, glucose, ketones, occult blood, specific gravity, and a microscopic examination. Screening of female participants included a serum pregnancy test (serum human chorionic gonadotrophin). A urine pregnancy test was performed at baseline and then at Months 6, 12, 18 and 24 for CR participants and at Months 12 and 24 only for controls. This test was also performed prior to the Hepatitis A vaccination and booster at Months 17 and 23. Results of all safety laboratory tests were reviewed by a clinical site physician for any abnormalities.

A standard resting 12-lead electrocardiogram (ECG) was recorded at screening, baseline and follow-up visits at month 1, 3, 6, 9, 12, 18, and 24 and was reviewed by the clinical site physician and a cardiologist. At screening or baseline, any candidate who had any of the following ECG abnormalities was considered ineligible for the study, and the screening process or baseline testing was terminated at that point. The abnormalities were signs of hyperkalemia, type II second or third degree heart block, ventricular ischemia, left bundle branch block, cardiac hypertrophy by any criteria, QRS complex > 100 ms in duration, abnormal QTc interval, superventricular tachycardia of any type not including atrial premature contractions, or ventricular arrhythmia of any type (including ventricular premature complexes more than 60 per minute), and exercise ECG recorded during the maximal oxygen uptake test demonstrating any of the above mentioned abnormalities occurring with exercise. The ECG protocol provided for discontinuation of the CR intervention if any of the ECG abnormalities, except for ventricular ischemia occurring with exercise, were observed at months 1, 3, 6, 9, 12, 18, and 24. Such participants would be asked to follow all other study procedures to the study end. Ventricular ischemia observed with the exercise in a participant enrolled in the CR group would result in temporary discontinuation of the intervention and a stress imaging study would be performed within two weeks. If a stress imaging study confirmed presence of ventricular ischemia, the CR intervention would be permanently discontinued and a participant would be asked to complete all outcome assessments to the study end.

There were seven safety surveillance protocols developed and implemented by CALERIE Safety Committee. The selection of the safety markers was informed by findings from CALERIE pilot studies. Participants were closely monitored for signs of hyperkalemia defined as increase in potassium levels above 5.5 mEq/L, significant drop in hematocrit below the lower limit of normal, cholesterol levels elevated above the upper limit of normal, eating disorders, depression and other mental or behavioral health conditions as well as excessive weight and bone loss. The protocols provided for the correction of these conditions and for temporary or permanent discontinuation of the CR intervention where indicated.

The study defined moderate depression as Beck Depression Inventory (BDI) score equal to or greater than 20 and less than 30; correspondingly, severe depression was defined as the BDI score of equal to or greater than 30.

Prior to the study, there were concerns about a possibility of excessive bone loss by CR participants. Correspondingly, the Safety Committee in consultation with the Data and Safety Monitoring Board and bone health experts implemented a comprehensive BMD monitoring protocol. In addition, all participants in both groups were provided with calcium and vitamin D supplements for two years. BMD at the hip, spine and forearm was measured with dual-energy X-ray absorptiometry (DXA). The hip and spine scans were obtained at baseline, 6, 12 and 24 months and were used to monitor participants for excessive bone loss (defined as BMD decrease of >10% at the total hip or lumbar spine). Scans were obtained using a standardized protocol for subject positioning, scan mode and analysis. All DXA measures were transmitted and assessed by a centralized reading center to maintain consistency and reduce variability.

Temporary discontinuation of the CR intervention was defined as cessation of the CR regimen for up to 30 days. The CR intervention was temporary discontinued when there was a significant increase in potassium level, or participant developed a treatment-resistant drop in hematocrit, depression or any other disease or condition where restriction in energy intake could interfere with the treatment or recovery (e.g., severe infections, recovery from trauma or surgery, and similar), or if there was a decrease in BMI <18.5 kg/m^2^. A number of newly diagnosed diseases and conditions such as cancer, persistent clinical drop in hematocrit, major clinical cardiovascular event, eating or psychiatric disorder, decrease in BMI below 18.5 kg/m^2^ or excessive bone loss (as defined above) and select clinical laboratory test abnormalities required permanent discontinuation of the CR intervention. Participants who permanently discontinued the CR intervention were strongly encouraged to continue the outcome measurements.

### Statistical methods

Simple descriptive statistics are used to summarize the characteristics of the study population. Between-group comparisons of the incidence of AEs and laboratory abnormalities were performed using Fisher's exact test. A similar approach was applied for differences between BMI strata within the CR group. All report p-values are nominal, not adjusted for multiple comparisons.
